# 4-Cyano-3-fluoro­phenyl 4-(hexa­dec­yl­oxy)benzoate

**DOI:** 10.1107/S1600536814001871

**Published:** 2014-02-05

**Authors:** M. K. Usha, H. T. Srinivas, Rajni Kant, Vivek K. Gupta, D. Revannasiddaiah

**Affiliations:** a Department of Studies in Physics, University of Mysore, Manasagangotri, Mysore 570 006, India; b Raman Research Institute, Bangalore 560 080, India; c X-ray Crystallography Laboratory, Post Graduate Department of Physics and Electronics, University of Jammu, Jammu Tawi 180 006, India

## Abstract

In the title compound, C_30_H_40_FNO_3_, the dihedral angle between the benzene rings is 57.76 (7)°. The alkyl chain adopts an all-*trans* conformation. In the crystal, mol­ecules are linked by pairs of C—H⋯O hydrogen bonds, forming inversion dimers.

## Related literature   

For general background to the title compound and applications of fluorinated liquid crystals, see: Chigrinov *et al.* (2008 ▶); Reddy & Tschierske (2006 ▶); Hird & Toyne (1998 ▶); Roussel (1999 ▶). For a related structure, see: Al-Eryani *et al.* (2011 ▶).
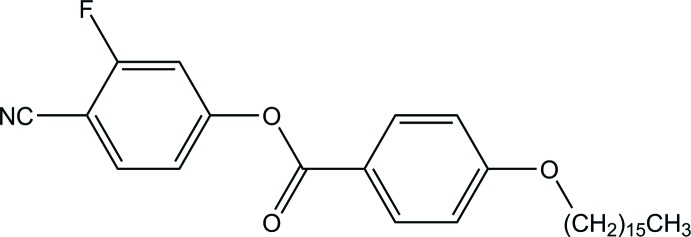


## Experimental   

### 

#### Crystal data  

C_30_H_40_FNO_3_
*M* *_r_* = 481.63
Monoclinic, 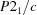

*a* = 22.937 (3) Å
*b* = 9.2022 (9) Å
*c* = 13.2859 (10) Å
β = 100.749 (8)°
*V* = 2755.1 (5) Å^3^
*Z* = 4
Mo *K*α radiation
μ = 0.08 mm^−1^
*T* = 293 K
0.30 × 0.20 × 0.20 mm

#### Data collection  

Oxford Diffraction Xcalibur Sapphire3 diffractometer
Absorption correction: multi-scan (*CrysAlis PRO*; Oxford Diffraction, 2010 ▶) *T*_min_ = 0.596, *T*_max_ = 0.985
10771 measured reflections
5379 independent reflections
2448 reflections with *I* > 2σ(*I*)
*R*_int_ = 0.047

#### Refinement  

*R*[*F* ^2^ > 2σ(*F* ^2^)] = 0.059
*wR*(*F* ^2^) = 0.146
*S* = 0.96
5379 reflections
318 parameters
H-atom parameters constrained
Δρ_max_ = 0.18 e Å^−3^
Δρ_min_ = −0.12 e Å^−3^

### 

Data collection: *CrysAlis PRO* (Oxford Diffraction, 2010 ▶); cell refinement: *CrysAlis PRO*; data reduction: *CrysAlis RED* (Oxford Diffraction, 2010 ▶); program(s) used to solve structure: *SHELXS97* (Sheldrick, 2008 ▶); program(s) used to refine structure: *SHELXL97* (Sheldrick, 2008 ▶); molecular graphics: *Mercury* (Macrae *et al.*, 2008 ▶); software used to prepare material for publication: *PLATON* (Spek, 2009 ▶).

## Supplementary Material

Crystal structure: contains datablock(s) global, I. DOI: 10.1107/S1600536814001871/is5333sup1.cif

Structure factors: contains datablock(s) I. DOI: 10.1107/S1600536814001871/is5333Isup2.hkl

Supporting information file. DOI: 10.1107/S1600536814001871/is5333Isup3.cml

CCDC reference:

Additional supporting information:  crystallographic information; 3D view; checkCIF report

## Figures and Tables

**Table 1:** Hydrogen-bond geometry (Å, °)

*D*—H⋯*A*	*D*—H	H⋯*A*	*D*⋯*A*	*D*—H⋯*A*
C5—H5⋯O10^i^	0.93	2.38	3.237 (3)	153

Symmetry code: (i) 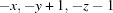.

**Fig. 1.**
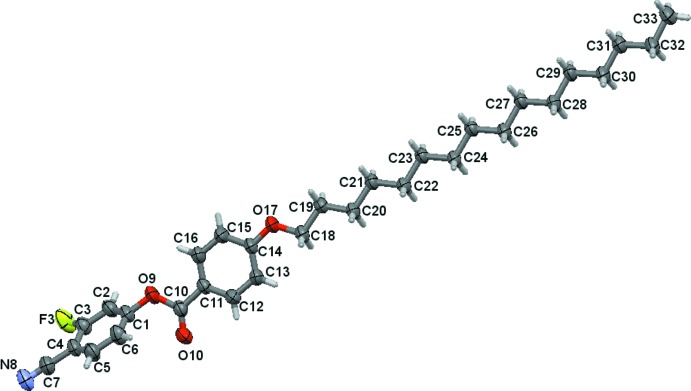
ORTEP diagram of the title compound with 50% probability ellipsoids.

**Fig. 2.**
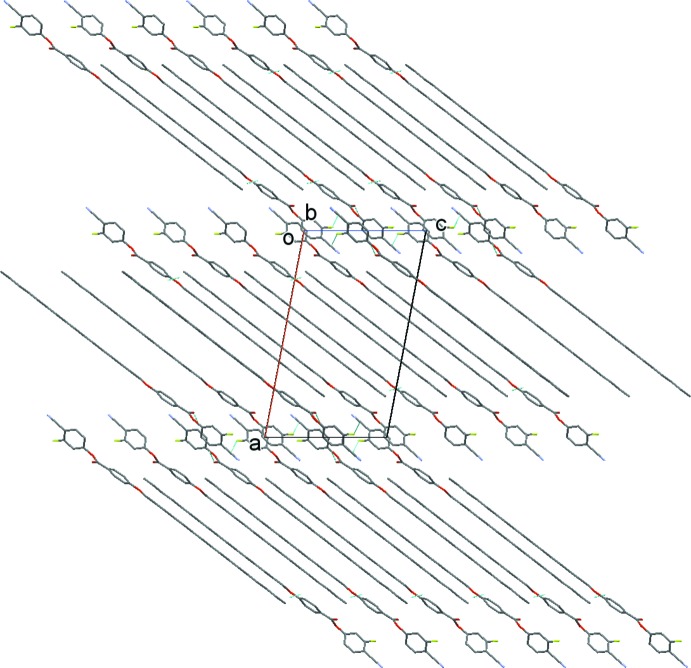
The packing arrangement of molecules viewed along the b axis. H atoms have been omitted for clarity

## supplementary crystallographic information

### 1. Comment

Low molar mass liquid crystals possessing low melting temperatures with good thermal range of liquid crystalline phase are in great demand for their potential applications such as, electro-optic display devices, optical switches, semiconductors, light modulators, electrically switchable color-tunable reflectors (Chigrinov *et al.*, 2008; Reddy & Tschierske, 2006). Partially fluorinated liquid crystals, owing to their low viscosity, high chemical and photochemical stability, high resistivity and positive dielectric anisotropy (generated by the high polarity of the C—F bond) are highly suited for the construction of active matrix thin film transistor (TFT) displays (Hird & Toyne, 1998; Roussel, 1999). With this background, we have synthesized the title compound, a novel low molar mass and fluorinated liquid crystal and herewith we report its crystal structure.

The *ORTEP* diagram of the title compound is shown (Fig. 1). The geometry of the molecule is similar to related structure of 4-(benzyloxy)phenyl 4-hexadecyloxy-3-methoxybenzoate (Al-Eryani *et al.*, 2011). In the title compound, the two benzene rings make a dihedral angle of 57.76 (7)°. An intermolecular C—H···O hydrogen bond (Table 1) links the molecules into a dimer (Fig. 2).

### 2. Experimental

A mixture of 2-fluoro-4-hydroxybenzonitrile (0.137 g, 1 equiv), 4-(hexadecyloxy) benzoic acid (0.362 g, 1 equiv) and 4-dimethylamino pyridine (DMAP) catalytic quantity was stirred in dry CH_2_Cl_2_. To the above clear solution, *N*,*N*-dicyclohexyl carbodiimide (DCC) (0.250 g, 1.2 equiv) was added and stirred for 30 minutes at room temperature. Dicyclohexylurea precipitate was filtered off and washed thoroughly with dry CH_2_Cl_2_. The combined filtrates were washed with water and dried over Na_2_SO_4_. The crude product was purified by column chromatography using silica gel (60–120 mesh) with 5% dichloromethane-hexane as eluent. The afforded white product was further purified by recrystallization with acetonitrile. This compound is found to exhibit liquid crystalline phase which has been confirmed using optical polarizing microscope and DSC. IR: 2920, 2850, 2233, 1741, 1602, 1454, 1247, 1107, 1045, 844 cm^-1^; ^1^H NMR (CDCl_3_): δ (p.p.m.) = 8.11 (m, 2H, Ar—H), 7.69 (m, 1H, Ar—H), 7.19 (m, 2H, Ar—H), 6.98 (m, 2H, Ar—H), 4.05 (t, 2H, –OCH_2_-, J = 6.55 Hz), 1.83–1.20 (m, 28H, –CH_2_-), 0.88 (s, 3H, –CH_3_).

### 3. Refinement

All the H atoms were positioned geometrically and were refined as riding on their parent C atoms, with C—H distances of 0.93–0.97 Å, and with *U*_iso_(H) = 1.2*U*_eq_(C) except for the methyl group where *U*_iso_(H) = 1.5*U*_eq_(C).

### Crystal data

**Table:**

C_30_H_40_FNO_3_	*F*(000) = 1040
*M**_r_* = 481.63	*D*_x_ = 1.161 Mg m^−^^3^
Monoclinic, *P*2_1_/*c*	Mo *K*α radiation, λ = 0.71073 Å
Hall symbol: -P 2ybc	Cell parameters from 2658 reflections
*a* = 22.937 (3) Å	θ = 3.9–28.9°
*b* = 9.2022 (9) Å	µ = 0.08 mm^−^^1^
*c* = 13.2859 (10) Å	*T* = 293 K
β = 100.749 (8)°	Block, white
*V* = 2755.1 (5) Å^3^	0.30 × 0.20 × 0.20 mm
*Z* = 4	

### Data collection

**Table:**

Oxford Diffraction Xcalibur Sapphire3 diffractometer	5379 independent reflections
Radiation source: fine-focus sealed tube	2448 reflections with *I* > 2σ(*I*)
Graphite monochromator	*R*_int_ = 0.047
Detector resolution: 16.1049 pixels mm^-1^	θ_max_ = 26.0°, θ_min_ = 3.6°
ω scans	*h* = −28→27
Absorption correction: multi-scan (*CrysAlis PRO*; Oxford Diffraction, 2010)	*k* = −11→10
*T*_min_ = 0.596, *T*_max_ = 0.985	*l* = −15→16
10771 measured reflections	

### Refinement

**Table:**

Refinement on *F*^2^	Secondary atom site location: difference Fourier map
Least-squares matrix: full	Hydrogen site location: inferred from neighbouring sites
*R*[*F*^2^ > 2σ(*F*^2^)] = 0.059	H-atom parameters constrained
*wR*(*F*^2^) = 0.146	*w* = 1/[σ^2^(*F*_o_^2^) + (0.0429*P*)^2^] where *P* = (*F*_o_^2^ + 2*F*_c_^2^)/3
*S* = 0.96	(Δ/σ)_max_ = 0.001
5379 reflections	Δρ_max_ = 0.18 e Å^−^^3^
318 parameters	Δρ_min_ = −0.12 e Å^−^^3^
0 restraints	Extinction correction: *SHELXL97* (Sheldrick, 2008), Fc^*^=kFc[1+0.001xFc^2^λ^3^/sin(2θ)]^-1/4^
Primary atom site location: structure-invariant direct methods	Extinction coefficient: 0.0023 (4)

### Special details

**Table:**

Geometry. Bond distances, angles *etc*. have been calculated using the rounded fractional coordinates. All su's are estimated from the variances of the (full) variance-covariance matrix. The cell e.s.d.'s are taken into account in the estimation of distances, angles and torsion angles
Refinement. Refinement on *F*^2^ for ALL reflections except those flagged by the user for potential systematic errors. Weighted *R*-factors *wR* and all goodnesses of fit *S* are based on *F*^2^, conventional *R*-factors *R* are based on *F*, with *F* set to zero for negative *F*^2^. The observed criterion of *F*^2^ > σ(*F*^2^) is used only for calculating -*R*-factor-obs *etc*. and is not relevant to the choice of reflections for refinement. *R*-factors based on *F*^2^ are statistically about twice as large as those based on *F*, and *R*-factors based on ALL data will be even larger.

### Fractional atomic coordinates and isotropic or equivalent isotropic displacement parameters (Å^2^)

**Table:**

	*x*	*y*	*z*	*U*_iso_*/*U*_eq_	
F3	0.01653 (7)	0.95837 (17)	−0.72555 (9)	0.0945 (7)	
O9	0.08886 (8)	0.82686 (18)	−0.37972 (10)	0.0720 (7)	
O10	0.11933 (8)	0.59458 (19)	−0.37524 (11)	0.0774 (7)	
O17	0.25399 (8)	0.84443 (17)	0.05228 (10)	0.0698 (6)	
N8	−0.10909 (11)	0.7467 (3)	−0.81803 (16)	0.0974 (11)	
C1	0.04873 (11)	0.8036 (3)	−0.47032 (16)	0.0588 (9)	
C2	0.05447 (11)	0.8891 (3)	−0.55275 (16)	0.0643 (10)	
C3	0.01283 (11)	0.8727 (3)	−0.64102 (16)	0.0621 (10)	
C4	−0.03311 (11)	0.7750 (3)	−0.65010 (16)	0.0583 (9)	
C5	−0.03811 (12)	0.6914 (3)	−0.56497 (17)	0.0714 (10)	
C6	0.00309 (12)	0.7071 (3)	−0.47546 (17)	0.0712 (11)	
C7	−0.07573 (12)	0.7596 (3)	−0.74352 (18)	0.0699 (10)	
C10	0.12016 (11)	0.7104 (3)	−0.33365 (16)	0.0560 (9)	
C11	0.15403 (10)	0.7469 (2)	−0.23164 (14)	0.0504 (8)	
C12	0.18556 (11)	0.6385 (3)	−0.17480 (15)	0.0602 (9)	
C13	0.21936 (11)	0.6657 (2)	−0.07876 (15)	0.0585 (9)	
C14	0.22127 (11)	0.8039 (2)	−0.03907 (15)	0.0540 (8)	
C15	0.18824 (11)	0.9125 (3)	−0.09427 (15)	0.0694 (10)	
C16	0.15494 (11)	0.8840 (3)	−0.18988 (15)	0.0650 (10)	
C18	0.28506 (11)	0.7371 (2)	0.11804 (14)	0.0562 (9)	
C19	0.32012 (11)	0.8146 (2)	0.20936 (14)	0.0551 (8)	
C20	0.35225 (10)	0.7152 (2)	0.29200 (14)	0.0546 (8)	
C21	0.38807 (11)	0.7963 (2)	0.38133 (14)	0.0546 (9)	
C22	0.42129 (11)	0.7045 (2)	0.46717 (14)	0.0541 (8)	
C23	0.45679 (11)	0.7898 (2)	0.55487 (14)	0.0521 (8)	
C24	0.49090 (10)	0.7010 (2)	0.64152 (13)	0.0531 (8)	
C25	0.52575 (10)	0.7893 (2)	0.72853 (13)	0.0523 (8)	
C26	0.56051 (10)	0.7018 (2)	0.81627 (14)	0.0540 (8)	
C27	0.59459 (10)	0.7921 (2)	0.90214 (14)	0.0543 (8)	
C28	0.62995 (11)	0.7061 (2)	0.99015 (14)	0.0548 (9)	
C29	0.66382 (11)	0.7978 (2)	1.07614 (14)	0.0579 (9)	
C30	0.69986 (11)	0.7133 (2)	1.16370 (14)	0.0600 (9)	
C31	0.73187 (11)	0.8036 (2)	1.25108 (15)	0.0601 (9)	
C32	0.76864 (11)	0.7210 (3)	1.33800 (15)	0.0662 (10)	
C33	0.80091 (12)	0.8162 (3)	1.42314 (16)	0.0834 (11)	
H2	0.08530	0.95560	−0.54900	0.0770*	
H5	−0.06900	0.62510	−0.56830	0.0860*	
H6	−0.00030	0.65150	−0.41840	0.0860*	
H12	0.18420	0.54470	−0.20130	0.0720*	
H13	0.24060	0.59100	−0.04150	0.0700*	
H15	0.18840	1.00550	−0.06690	0.0830*	
H16	0.13290	0.95810	−0.22650	0.0780*	
H18A	0.31150	0.68300	0.08270	0.0670*	
H18B	0.25730	0.66960	0.13970	0.0670*	
H19A	0.29340	0.87620	0.23890	0.0660*	
H19B	0.34900	0.87720	0.18620	0.0660*	
H20A	0.32340	0.65430	0.31670	0.0660*	
H20B	0.37850	0.65210	0.26260	0.0660*	
H21A	0.41650	0.85740	0.35560	0.0660*	
H21B	0.36150	0.86010	0.40950	0.0660*	
H22A	0.44800	0.64060	0.43950	0.0650*	
H22B	0.39300	0.64390	0.49370	0.0650*	
H23A	0.48460	0.85110	0.52790	0.0620*	
H23B	0.42990	0.85320	0.58240	0.0620*	
H24A	0.51820	0.63810	0.61440	0.0640*	
H24B	0.46320	0.63930	0.66870	0.0640*	
H25A	0.49840	0.85200	0.75550	0.0630*	
H25B	0.55320	0.85120	0.70110	0.0630*	
H26A	0.53320	0.63960	0.84390	0.0650*	
H26B	0.58820	0.63950	0.78960	0.0650*	
H27A	0.56680	0.85400	0.92890	0.0650*	
H27B	0.62160	0.85490	0.87430	0.0650*	
H28A	0.60300	0.64310	1.01800	0.0660*	
H28B	0.65790	0.64460	0.96360	0.0660*	
H29A	0.69030	0.86180	1.04790	0.0690*	
H29B	0.63570	0.85850	1.10310	0.0690*	
H30A	0.67360	0.64670	1.19010	0.0720*	
H30B	0.72890	0.65530	1.13700	0.0720*	
H31A	0.75760	0.87120	1.22430	0.0720*	
H31B	0.70270	0.86040	1.27820	0.0720*	
H32A	0.79760	0.66300	1.31120	0.0790*	
H32B	0.74300	0.65490	1.36630	0.0790*	
H33A	0.82760	0.87960	1.39650	0.1250*	
H33B	0.82300	0.75640	1.47610	0.1250*	
H33C	0.77260	0.87330	1.45090	0.1250*	

### Atomic displacement parameters (Å^2^)

**Table:**

	*U*^11^	*U*^22^	*U*^33^	*U*^12^	*U*^13^	*U*^23^
F3	0.1042 (14)	0.1239 (13)	0.0487 (8)	−0.0370 (11)	−0.0027 (7)	0.0190 (8)
O9	0.0806 (14)	0.0730 (11)	0.0487 (9)	−0.0020 (10)	−0.0230 (8)	−0.0015 (8)
O10	0.0708 (14)	0.0826 (13)	0.0679 (11)	0.0087 (11)	−0.0152 (9)	−0.0231 (9)
O17	0.0810 (14)	0.0649 (10)	0.0495 (9)	0.0109 (10)	−0.0241 (8)	−0.0055 (8)
N8	0.088 (2)	0.113 (2)	0.0735 (15)	0.0049 (17)	−0.0308 (13)	−0.0068 (14)
C1	0.0608 (18)	0.0662 (16)	0.0429 (13)	0.0007 (14)	−0.0068 (11)	−0.0036 (12)
C2	0.0612 (18)	0.0727 (18)	0.0522 (14)	−0.0081 (14)	−0.0068 (12)	−0.0039 (12)
C3	0.0663 (19)	0.0713 (17)	0.0440 (14)	−0.0041 (15)	−0.0018 (12)	0.0036 (12)
C4	0.0539 (17)	0.0689 (17)	0.0449 (14)	0.0024 (14)	−0.0097 (11)	−0.0017 (12)
C5	0.0604 (19)	0.0788 (19)	0.0674 (16)	−0.0109 (15)	−0.0075 (13)	0.0036 (14)
C6	0.068 (2)	0.084 (2)	0.0562 (16)	−0.0095 (17)	−0.0024 (13)	0.0126 (13)
C7	0.066 (2)	0.0752 (18)	0.0602 (16)	0.0038 (15)	−0.0095 (13)	0.0005 (13)
C10	0.0503 (16)	0.0657 (16)	0.0479 (14)	0.0009 (14)	−0.0018 (11)	−0.0016 (12)
C11	0.0476 (15)	0.0592 (15)	0.0401 (12)	−0.0033 (13)	−0.0032 (10)	−0.0006 (10)
C12	0.0632 (18)	0.0571 (15)	0.0538 (14)	0.0046 (14)	−0.0057 (12)	−0.0109 (11)
C13	0.0615 (18)	0.0545 (14)	0.0517 (14)	0.0057 (13)	−0.0097 (12)	0.0009 (11)
C14	0.0558 (16)	0.0604 (15)	0.0393 (12)	0.0032 (13)	−0.0081 (11)	−0.0006 (11)
C15	0.089 (2)	0.0554 (15)	0.0524 (14)	0.0084 (15)	−0.0160 (13)	−0.0069 (11)
C16	0.0714 (19)	0.0585 (16)	0.0545 (14)	0.0032 (15)	−0.0157 (12)	0.0017 (12)
C18	0.0574 (17)	0.0617 (15)	0.0437 (13)	0.0033 (13)	−0.0056 (11)	−0.0002 (11)
C19	0.0553 (16)	0.0613 (15)	0.0433 (12)	0.0034 (13)	−0.0049 (11)	−0.0031 (10)
C20	0.0601 (17)	0.0562 (14)	0.0414 (12)	0.0014 (13)	−0.0062 (11)	0.0006 (10)
C21	0.0573 (17)	0.0591 (15)	0.0420 (12)	0.0000 (13)	−0.0050 (11)	0.0013 (10)
C22	0.0601 (17)	0.0524 (14)	0.0443 (12)	0.0026 (13)	−0.0045 (11)	−0.0014 (10)
C23	0.0569 (16)	0.0528 (14)	0.0407 (12)	−0.0010 (12)	−0.0058 (10)	0.0006 (10)
C24	0.0580 (17)	0.0526 (14)	0.0430 (12)	0.0034 (12)	−0.0054 (11)	−0.0027 (10)
C25	0.0555 (16)	0.0528 (14)	0.0434 (12)	−0.0029 (12)	−0.0043 (11)	0.0007 (10)
C26	0.0582 (17)	0.0554 (14)	0.0424 (12)	0.0039 (13)	−0.0058 (11)	−0.0010 (10)
C27	0.0595 (17)	0.0551 (14)	0.0417 (12)	−0.0017 (13)	−0.0076 (11)	0.0024 (10)
C28	0.0586 (17)	0.0583 (15)	0.0415 (12)	0.0015 (13)	−0.0062 (11)	0.0019 (10)
C29	0.0602 (17)	0.0612 (15)	0.0449 (13)	−0.0007 (13)	−0.0091 (11)	0.0027 (11)
C30	0.0672 (19)	0.0622 (16)	0.0426 (13)	0.0079 (14)	−0.0105 (11)	−0.0003 (11)
C31	0.0609 (18)	0.0679 (16)	0.0457 (13)	0.0006 (14)	−0.0054 (11)	−0.0012 (11)
C32	0.0667 (19)	0.0728 (17)	0.0504 (14)	0.0111 (15)	−0.0114 (12)	−0.0042 (12)
C33	0.084 (2)	0.098 (2)	0.0563 (15)	0.0051 (18)	−0.0173 (14)	−0.0121 (13)

### Geometric parameters (Å, º)

**Table:**

F3—C3	1.388 (3)	C6—H6	0.9300
O9—C1	1.389 (3)	C12—H12	0.9300
O9—C10	1.369 (3)	C13—H13	0.9300
O10—C10	1.199 (3)	C15—H15	0.9300
O17—C14	1.355 (2)	C16—H16	0.9300
O17—C18	1.419 (2)	C18—H18A	0.9700
N8—C7	1.139 (3)	C18—H18B	0.9700
C1—C2	1.375 (3)	C19—H19A	0.9700
C1—C6	1.365 (4)	C19—H19B	0.9700
C2—C3	1.376 (3)	C20—H20A	0.9700
C3—C4	1.373 (4)	C20—H20B	0.9700
C4—C5	1.390 (3)	C21—H21A	0.9700
C4—C7	1.437 (3)	C21—H21B	0.9700
C5—C6	1.382 (3)	C22—H22A	0.9700
C10—C11	1.470 (3)	C22—H22B	0.9700
C11—C12	1.373 (3)	C23—H23A	0.9700
C11—C16	1.377 (3)	C23—H23B	0.9700
C12—C13	1.387 (3)	C24—H24A	0.9700
C13—C14	1.374 (3)	C24—H24B	0.9700
C14—C15	1.380 (3)	C25—H25A	0.9700
C15—C16	1.380 (3)	C25—H25B	0.9700
C18—C19	1.505 (3)	C26—H26A	0.9700
C19—C20	1.511 (3)	C26—H26B	0.9700
C20—C21	1.509 (3)	C27—H27A	0.9700
C21—C22	1.507 (3)	C27—H27B	0.9700
C22—C23	1.511 (3)	C28—H28A	0.9700
C23—C24	1.506 (3)	C28—H28B	0.9700
C24—C25	1.514 (3)	C29—H29A	0.9700
C25—C26	1.515 (3)	C29—H29B	0.9700
C26—C27	1.507 (3)	C30—H30A	0.9700
C27—C28	1.516 (3)	C30—H30B	0.9700
C28—C29	1.513 (3)	C31—H31A	0.9700
C29—C30	1.511 (3)	C31—H31B	0.9700
C30—C31	1.503 (3)	C32—H32A	0.9700
C31—C32	1.503 (3)	C32—H32B	0.9700
C32—C33	1.511 (3)	C33—H33A	0.9600
C2—H2	0.9300	C33—H33B	0.9600
C5—H5	0.9300	C33—H33C	0.9600
			
C1—O9—C10	118.32 (19)	C21—C20—H20A	109.00
C14—O17—C18	119.38 (16)	C21—C20—H20B	109.00
O9—C1—C2	117.1 (2)	H20A—C20—H20B	108.00
O9—C1—C6	121.5 (2)	C20—C21—H21A	108.00
C2—C1—C6	121.3 (2)	C20—C21—H21B	108.00
C1—C2—C3	117.5 (2)	C22—C21—H21A	108.00
F3—C3—C2	119.4 (2)	C22—C21—H21B	108.00
F3—C3—C4	117.56 (19)	H21A—C21—H21B	107.00
C2—C3—C4	123.1 (2)	C21—C22—H22A	109.00
C3—C4—C5	118.0 (2)	C21—C22—H22B	109.00
C3—C4—C7	121.9 (2)	C23—C22—H22A	109.00
C5—C4—C7	120.2 (2)	C23—C22—H22B	109.00
C4—C5—C6	119.7 (2)	H22A—C22—H22B	108.00
C1—C6—C5	120.4 (2)	C22—C23—H23A	108.00
N8—C7—C4	179.3 (3)	C22—C23—H23B	108.00
O9—C10—O10	121.9 (2)	C24—C23—H23A	108.00
O9—C10—C11	112.1 (2)	C24—C23—H23B	108.00
O10—C10—C11	126.0 (2)	H23A—C23—H23B	107.00
C10—C11—C12	118.35 (19)	C23—C24—H24A	109.00
C10—C11—C16	123.1 (2)	C23—C24—H24B	109.00
C12—C11—C16	118.51 (19)	C25—C24—H24A	109.00
C11—C12—C13	121.5 (2)	C25—C24—H24B	109.00
C12—C13—C14	119.5 (2)	H24A—C24—H24B	108.00
O17—C14—C13	124.83 (19)	C24—C25—H25A	108.00
O17—C14—C15	115.74 (18)	C24—C25—H25B	108.00
C13—C14—C15	119.4 (2)	C26—C25—H25A	108.00
C14—C15—C16	120.4 (2)	C26—C25—H25B	108.00
C11—C16—C15	120.6 (2)	H25A—C25—H25B	107.00
O17—C18—C19	107.34 (15)	C25—C26—H26A	109.00
C18—C19—C20	114.45 (16)	C25—C26—H26B	109.00
C19—C20—C21	113.10 (15)	C27—C26—H26A	109.00
C20—C21—C22	116.25 (16)	C27—C26—H26B	109.00
C21—C22—C23	114.59 (16)	H26A—C26—H26B	108.00
C22—C23—C24	115.84 (16)	C26—C27—H27A	109.00
C23—C24—C25	114.68 (15)	C26—C27—H27B	109.00
C24—C25—C26	115.43 (15)	C28—C27—H27A	109.00
C25—C26—C27	114.43 (15)	C28—C27—H27B	109.00
C26—C27—C28	115.06 (16)	H27A—C27—H27B	107.00
C27—C28—C29	114.63 (15)	C27—C28—H28A	109.00
C28—C29—C30	115.12 (16)	C27—C28—H28B	109.00
C29—C30—C31	115.37 (16)	C29—C28—H28A	109.00
C30—C31—C32	115.92 (17)	C29—C28—H28B	109.00
C31—C32—C33	114.1 (2)	H28A—C28—H28B	108.00
C1—C2—H2	121.00	C28—C29—H29A	108.00
C3—C2—H2	121.00	C28—C29—H29B	108.00
C4—C5—H5	120.00	C30—C29—H29A	109.00
C6—C5—H5	120.00	C30—C29—H29B	109.00
C1—C6—H6	120.00	H29A—C29—H29B	107.00
C5—C6—H6	120.00	C29—C30—H30A	108.00
C11—C12—H12	119.00	C29—C30—H30B	108.00
C13—C12—H12	119.00	C31—C30—H30A	108.00
C12—C13—H13	120.00	C31—C30—H30B	108.00
C14—C13—H13	120.00	H30A—C30—H30B	107.00
C14—C15—H15	120.00	C30—C31—H31A	108.00
C16—C15—H15	120.00	C30—C31—H31B	108.00
C11—C16—H16	120.00	C32—C31—H31A	108.00
C15—C16—H16	120.00	C32—C31—H31B	108.00
O17—C18—H18A	110.00	H31A—C31—H31B	107.00
O17—C18—H18B	110.00	C31—C32—H32A	109.00
C19—C18—H18A	110.00	C31—C32—H32B	109.00
C19—C18—H18B	110.00	C33—C32—H32A	109.00
H18A—C18—H18B	109.00	C33—C32—H32B	109.00
C18—C19—H19A	109.00	H32A—C32—H32B	108.00
C18—C19—H19B	109.00	C32—C33—H33A	109.00
C20—C19—H19A	109.00	C32—C33—H33B	110.00
C20—C19—H19B	109.00	C32—C33—H33C	109.00
H19A—C19—H19B	108.00	H33A—C33—H33B	109.00
C19—C20—H20A	109.00	H33A—C33—H33C	109.00
C19—C20—H20B	109.00	H33B—C33—H33C	110.00
			
C10—O9—C1—C2	−126.7 (3)	C10—C11—C12—C13	178.8 (2)
C10—O9—C1—C6	57.7 (3)	C16—C11—C12—C13	−2.1 (4)
C1—O9—C10—O10	9.4 (3)	C10—C11—C16—C15	−179.1 (2)
C1—O9—C10—C11	−171.6 (2)	C12—C11—C16—C15	1.9 (4)
C18—O17—C14—C13	−5.9 (3)	C11—C12—C13—C14	0.3 (4)
C18—O17—C14—C15	174.4 (2)	C12—C13—C14—O17	−178.0 (2)
C14—O17—C18—C19	176.4 (2)	C12—C13—C14—C15	1.7 (4)
O9—C1—C2—C3	−176.3 (2)	O17—C14—C15—C16	177.8 (2)
C6—C1—C2—C3	−0.7 (4)	C13—C14—C15—C16	−2.0 (4)
O9—C1—C6—C5	176.6 (2)	C14—C15—C16—C11	0.1 (4)
C2—C1—C6—C5	1.1 (4)	O17—C18—C19—C20	175.26 (19)
C1—C2—C3—F3	179.1 (2)	C18—C19—C20—C21	178.7 (2)
C1—C2—C3—C4	−0.6 (4)	C19—C20—C21—C22	179.8 (2)
F3—C3—C4—C5	−178.3 (2)	C20—C21—C22—C23	179.8 (2)
F3—C3—C4—C7	0.7 (4)	C21—C22—C23—C24	−179.5 (2)
C2—C3—C4—C5	1.4 (4)	C22—C23—C24—C25	−179.67 (19)
C2—C3—C4—C7	−179.6 (3)	C23—C24—C25—C26	−179.82 (19)
C3—C4—C5—C6	−0.9 (4)	C24—C25—C26—C27	−179.73 (19)
C7—C4—C5—C6	−180.0 (2)	C25—C26—C27—C28	−179.58 (19)
C4—C5—C6—C1	−0.3 (4)	C26—C27—C28—C29	−179.8 (2)
O9—C10—C11—C12	177.3 (2)	C27—C28—C29—C30	−179.3 (2)
O9—C10—C11—C16	−1.7 (3)	C28—C29—C30—C31	−177.9 (2)
O10—C10—C11—C12	−3.7 (4)	C29—C30—C31—C32	−179.1 (2)
O10—C10—C11—C16	177.3 (2)	C30—C31—C32—C33	178.9 (2)

### Hydrogen-bond geometry (Å, º)

**Table:**

*D*—H···*A*	*D*—H	H···*A*	*D*···*A*	*D*—H···*A*
C5—H5···O10^i^	0.93	2.38	3.237 (3)	153

Symmetry code: (i) −*x*, −*y*+1, −*z*−1.
